# Bub1 autophosphorylation feeds back to regulate kinetochore docking and promote localized substrate phosphorylation

**DOI:** 10.1038/ncomms9364

**Published:** 2015-09-24

**Authors:** Adeel Asghar, Audrey Lajeunesse, Kalyan Dulla, Guillaume Combes, Philippe Thebault, Erich A. Nigg, Sabine Elowe

**Affiliations:** 1 Faculty of Medicine, Department of Molecular and Cellular Biology, Université Laval, Québec, Canada G1V 0A6; 2 Department of Reproduction, Mother and Youth Health, Centre de recherche du Centre Hospitalier Universitaire de Québec, Québec, Canada G1V 4G2; 3 ProQR Therapeutics N.V., Darwinweg 24, Leiden 2333 CR, The Netherlands; 4 Biozentrum, University of Basel, Klingelbergstrasse 50/70, Basel CH-4056, Switzerland

## Abstract

During mitosis, Bub1 kinase phosphorylates histone H2A-T120 to promote centromere sister chromatid cohesion through recruitment of shugoshin (Sgo) proteins. The regulation and dynamics of H2A-T120 phosphorylation are poorly understood. Using quantitative phosphoproteomics we show that Bub1 is autophosphorylated at numerous sites. We confirm mitosis-specific autophosphorylation of a several residues and show that Bub1 activation is primed in interphase but fully achieved only in mitosis. Mutation of a single autophosphorylation site T589 alters kinetochore turnover of Bub1 and results in uniform H2A-T120 phosphorylation and Sgo recruitment along chromosome arms. Consequently, improper sister chromatid resolution and chromosome segregation errors are observed. Kinetochore tethering of Bub1-T589A refocuses H2A-T120 phosphorylation and Sgo1 to centromeres. Recruitment of the Bub1-Bub3-BubR1 axis to kinetochores has recently been extensively studied. Our data provide novel insight into the regulation and kinetochore residency of Bub1 and indicate that its localization is dynamic and tightly controlled through feedback autophosphorylation.

## Abstract (web summary)

Bub1 kinase phosphorylates histone H2A-T120 at the centromere to recruit shugoshin proteins and promote sister chromatid cohesion during mitosis. Here the authors show that Bub1 autophosphorylation on T589 influences Bub1 dynamics at the kinetochore and restricts H2A-T120 phosphorylation to centromeres.

## Graphical abstract

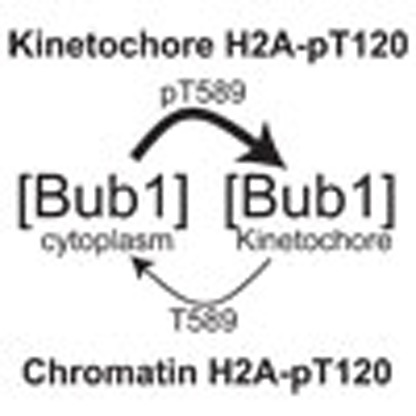

The accurate traverse through mitosis results in equal allocation of duplicated sister chromosomes and is critical for cellular and organism health. To ensure this, eukaryotes have evolved a safeguard mechanism known as the spindle assembly checkpoint (SAC), which functions during both meiosis and mitosis^1^,^2^,^3^,^4^,^5^, and monitors the correct attachment of kinetochores to microtubules. The activities of both the SAC and the microtubule attachment machinery are orchestrated by a network of kinases and phosphatases. SAC kinases including budding uninhibited by benzamidazole 1 (Bub1), monopolar spindle 1 (Mps1) and Aurora B play a dual and interconnected role in microtubule attachment regulation and SAC signalling^6^,^7^. Recently, a remarkable body of work has begun to outline how these kinases (and their counteracting phosphatases) monitor the status of attachments and relay this as a diffusible biochemical signal. A clear picture of the recruitment of the checkpoint kinase Bub1 to the kinetochore is beginning to emerge. Mps1 phosphorylation of so-called MELT motifs on the KNL1 subunit of the macromolecular KMN complex together with the KI (Lys-Ile) motifs of KNL1 promote the recruitment of Bub1–Bub3 in a manner that involves multiple cooperative interactions^5^,^8^. Less well understood is how this recruitment is dynamically regulated, although recent evidence supports a role for the protein phosphatases PP2A and PP1 in determining the extent of Bub1 recruitment^9^,^10^. The current model posits that once at the kinetochore, Bub1 acts as a stable scaffold for recruitment of anaphase promoting complex/cyclosome (APC/C) inhibitors including BubR1, Mad1 and Mad2, as well as centromere proteins E and F, and the mitotic centromere-associated kinesin; this scaffolding function of Bub1 is thought to be kinase independent^6^,^11^,^12^.

Bub1 also has kinase-dependent functions during mitosis. Cdc20 is an *in vitro* target of Bub1 and this phosphorylation may directly contribute to APC/Cdc20 inhibition^13^. Bub1 phosphorylation of the conserved histone H2A at T120 (H2A-T120, human numbering) results in a histone mark that mediates the recruitment of MEI-S332/shugoshin (Sgo) proteins to the centromere during both meiosis and mitosis^14^. In mammalian mitosis, Bub1 recruitment of Sgo1 in complex with protein phosphatase 2A protects cohesion at centromeres until the metaphase–anaphase transition^15^,^16^,^17^,^18^. The kinase activity of Bub1 is therefore clearly critical for ensuring faithful chromosome segregation and recent elegant work has begun to elucidate how Bub1 kinase activity is regulated. Crystal structures and biochemical studies have shown that autophosphorylation of Bub1 in the activation segment results in conformational changes of this region to selectively regulate the activity of Bub1 towards H2A-T120 (ref. 19). Thus, another important substrate of Bub1 is Bub1 itself.

Here we use a quantitative proteomics approach to identify Bub1-specific autophosphorylation sites. We show that Bub1 is significantly autophosphorylated outside the activation segment and kinase domain, including at the conserved threonine 589 (T589). We show the Bub1 activity is primed in interphase but does not fully mature until mitosis. Immunofluorescence with a phosphospecific antibody indicates that autophosphorylation at T589 is prevalent during early mitosis. Alanine substitution of this residue (T589A) results in chromosome missegregation and incomplete sister chromatid arm resolution as a result of non-localized H2A-T120 phosphorylation and ectopic Sgo1 recruitment. Fluorescence recovery after photobleaching (FRAP) experiments reveal that Bub1-T589A and Bub1-kinase dead (D946A, hereafter referred to as KD) exhibit more rapid kinetochore turnover than wild-type (WT) protein. Forced localization of Bub1-T589A to the kinetochore (KT) refocuses H2A-T120 phosphorylation and Sgo1 localization to the kinetochore. We propose that spatially constrained H2A-T120 phosphorylation, and thus sister chromatid cohesion, are promoted by a positive feedback mechanism formed by autophosphorylation of Bub1 at T589 that regulates the dynamics of Bub1 kinetochore docking.

## Results

### Identification of Bub1 autophosphorylation sites

**Figure 1:**
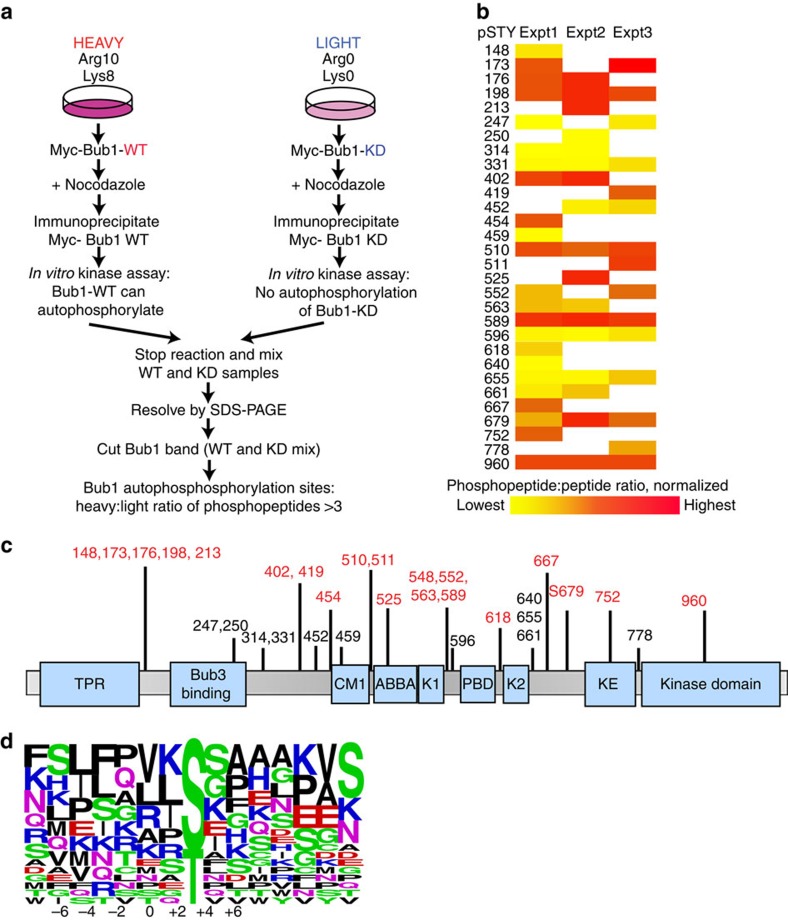
Identification of Bub1 autophosphorylation sites. (**a**) Schematic of the SILAC protocol for identification of Bub1 autophosphorylation sites. (**b**) Heat map representation of normalized phosphopeptide:peptide ratio of phosphosites identified on Bub1 from three independent MS experiments. (**c**) Cartoon illustration of the position of the identified phosphorylation sites relative to the functional domains of Bub1. Autophosphorylation sites are red; other phosphorylation sites are in black. (**d**) Weblogo representation and amino acid enrichment of Bub1 surrounding phosphorylation sites.

To identify Bub1 autophosphorylation sites, we devised an approach based on stable isotope labelling in cell culture (SILAC, Fig. 1a) of Bub1 WT and KD. To enable quantitation of the changes of phosphopeptide abundance by mass spectrometry (MS), cells were labelled by growing them in medium containing either light arginine and lysine (Arg0/Lys0) or the heavy isotopic variants [^13^C_6_,^15^N_4_]arginine and [^13^C_6_,^15^N_2_]lysine (Arg10/Lys8). Immunoprecipitated, mitotic, Bub1-WT and Bub1-KD expressed in differentially labelled cells were separately subjected to a non-radioactive *in-vitro* kinase assay. This autophosphorylation amplification step was introduced to increase the occupancy of phosphorylation sites within Bub1 and thus increase phosphopeptide detection and, importantly, to allow distinction between genuine autophosphorylation sites and phosphorylation incurred by co-precipitating kinases. We also considered this approach superior to an *in-vitro* assay of recombinant proteins as Bub1 mitotic modifications, localization and binding partners may all contribute to genuine and physiologically relevant Bub1 autophosphorylation. The experiment was performed in triplicate with minor changes: the amino acid labelling was reversed in one replicate (exp2, Fig. 1b) to control for a potential effect of amino acid labelling, and in the final replicate (exp3, Fig. 1b) a combination of Lys-C, Glu-C and elastase were used instead of trypsin to diversify peptide coverage.

Data from the three independent experiments resulted in a combined coverage of 68% of Bub1 and a total of 38 unique phosphorylation sites (MASCOT score cutoff of ≥13; Class I sites^20^ (Supplementary Table S1), of which 30 sites could be definitively assigned SILAC and protein ratios in at least 1 replicate. Threonine 960 phosphorylation in the activation segment of the kinase domain was identified in all three experiments, was found to have a high phosphopeptide:peptide ratio and was used as reference for normalization, results of which are shown in Fig. 1b. Several additional phosphosites were identified after Lys-C, Glu-C and elastase digestion, but contained neither lysine nor arginine and no SILAC ratio could be assigned. These were thus excluded from further analysis (see Supplementary Data 1).

Of the sites we identified, 19 were novel, whereas 19 have been previously curated in PhosphoSitePlus. The majority of the phosphorylation sites identified were situated in low complexity stretches in between domains (Fig. 1c), with the exception of T960 in the kinase activation segment.

Twenty phosphosites were significantly upregulated in Bub1-WT compared with Bub1-KD and thus considered potential Bub1 autophosphorylation sites (Fig. 1c, red). These sites exhibited a fold increase in phosphopeptide:peptide ratio of ≥3, considered a conservative cutoff requirement for fold change^21^. Alignment of these sites, together with H2A-T120 (Fig. 1d), suggested a tendency for basic (mainly *K* at positions −1 and +5) and small non-polar (at positions +2 and +3) residues relative to the phosphoacceptor, as well as an exclusion of acidic residues surrounding the phosphosites. Of the phosphosites that were not considered to be Bub1 dependent (phosphopeptide:peptide ratio <3), 50% (residues 452, 459, 596 and 655) were followed by a proline, suggesting that they may be targets of a proline-directed kinase such as CDK1 or mitogen-activated protein kinase, in agreement with previous observations^22^,^23^,^24^. S314 and S331 adhered to the consensus for ATM/ATR kinases; S314 was previously identified as an ATM site and may be required for Bub1 activation^25^,^26^. Two additional sites, S247 and S250, adhered to a Plk1/Mps1 consensus, which have also been shown to phosphorylate Bub1 (refs 24, 27). Thus, Bub1 is highly phosphorylated by a number of mitotic kinases, including itself.

### Regulation of Bub1 activation and autophosphorylation

**Figure 2:**
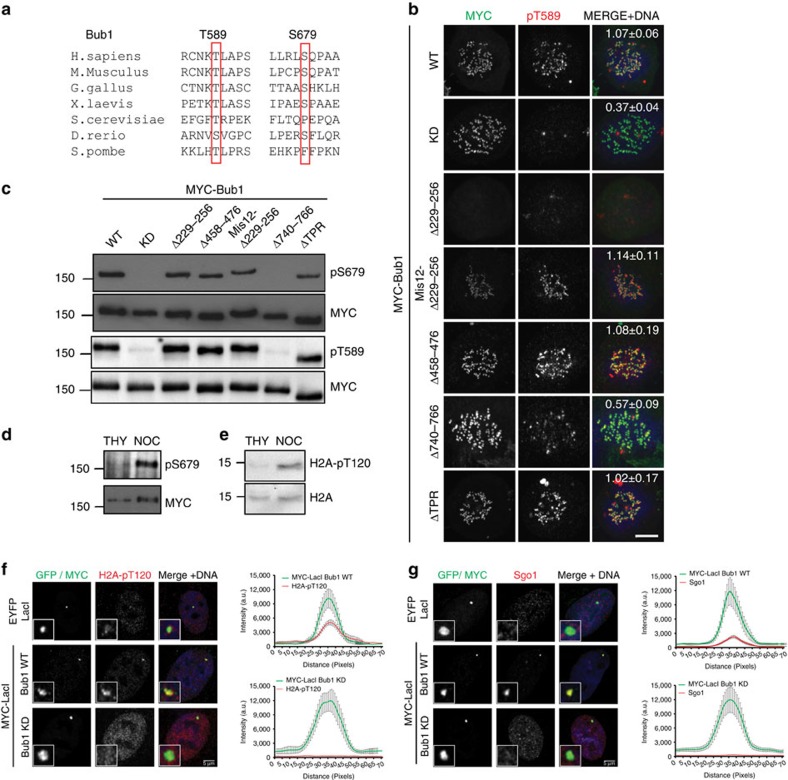
Full Bub1 activation is mitotic specific and requires the kinase extension domain. (**a**) Evolutionary conservation of Bub1 T589 and S679. (**b**) Bub1 deletion mutants were expressed in HeLa cells depleted of endogenous Bub1. Mitotic cells were stained with Hoechst (Blue in merge), anti-MYC (green) and anti-pT589 (red). Scale bar, 10 μM. Quantitation of pT589 signal relative to CREST at kinetochores (mean±s.e.) from a minimum of ten cells per condition is indicated in the right-most panel. (**c**) Cells were transfected with Bub1 mutants as in **b** and enriched in mitosis by nocodazole treatment. Anti-pT679 (upper half) and anti-pT589 (bottom half) western blottings were performed with MYC-Bub1 immunoprecipitated from equalized lysates. Anti-MYC blotting (second and fourth panels) reveals equal loading. (**d**) MYC-Bub1 was immunoprecipiated from HeLa cells stably expressing MYC-Bub1-WT arrested in G1/S or mitosis by thymidine (THY) or nocodazole (NOC) treatment, respectively, and blotted with anti-pT679 antibodies (upper panel) or stripped and reprobed with anti-MYC. (**e**) Western blottings of histones purified from thymidine- and nocodazole-arrested cells with anti-H2A-pT120 (upper panel) and anti-H2A (lower panel) antibodies. (**f**,**g**) U2OS cells expressing a 256-copy array of the *lac* operator were transfected with a LacI-GFP, 3XMYC-LacI-Bub1-WT or KD. Fixed cells were stained with Hoechst (blue), anti-MYC or GFP in the control (green) and either anti-H2A-pT120 (red, **f**) or anti-Sgo1 (red, **g**). The overlap between the MYC and H2A-pT120 or Sgo1 is shown in the panel on the right of each figure. Error bars represent s.e. Scale bar, 5 μM.

To investigate Bub1 autophosphorylation at sites outside the activation segment, we generated phosphospecific antibodies towards two potential autophosphorylation sites, T589 and S679. These sites (see Fig. 2a for evolutionary alignment) were consistently autophosphorylated in our MS experiments. They were also preceded by at least one basic residue at the −1 (T589) or −2 (S679) position and have been independently observed in large-scale mitotic MS screens^21^,^28^. We thus reasoned that they were genuine *in vivo* autophosphorylation sites. Anti-pT589 staining of fixed cells clearly decorated kinetochores and overlapped the Bub1 signal in prophase and prometaphase (Supplementary Fig. 1a). Anti-pT589 signal was lost on depletion of Bub1 or phosphatase treatment (Supplementary Fig. 1b), demonstrating that the pT589 signal is both Bub1 dependent and phosphospecific. Importantly, depletion and rescue experiments revealed that the pT589 signal was lost in Bub1-KD and Bub1-T589A-expressing cells (Supplementary Fig. 1c), indicating that phosphorylation at T589 is strictly dependent on Bub1 kinase activity, in agreement with its identification as an autophosphorylation site. No signal was detected by immunofluorescence with anti-pS679 antibody, although there was a clear signal on western blottings. Anti-pS679 detects Bub1 from mitotic extracts, before but not after phosphatase treatment, demonstrating phosphospecificity of this antibody (Supplementary Fig. 1d).

A number of groups have recently reported on the role of the Bub1 tetratricopeptide repeat (TPR) domain in regulating kinase activity with conflicting results^19^,^29^,^30^. We thus sought to determine the domains of Bub1 required for kinase function as measured by autophosphorylation. We depleted endogenous Bub1 with small interfering RNAs (siRNAs) targeting the 3′-untranslated region (3′-UTR)^31^ and expressed MYC-tagged Bub1, WT, KD, the Bub3-binding mutant (Δ229–256), the checkpoint mutant in conserved motif I (Δ458–476), the kinase extension domain mutant (Δ740–766)^12^ and the ΔTPR in HeLa cells. We then determined phosphorylation at T589 (Fig. 2b,c) and S679 (Fig. 2c). As the Bub3-binding mutant Δ229–256 does not bind to the kinetochore, we forced kinetochore localization using a Mis12-tag to determine the role of Bub3 binding in Bub1 activation independent of its role in kinetochore recruitment. As expected, Bub1-WT-expressing cells demonstrated robust pT589 and pS679 signal, whereas little or no signal was observed in cells expressing Bub1-KD or the Bub1 kinase extension domain mutant (Δ740–766, Fig. 2b,c), confirming the status of these sites as *bona fide* Bub1 autophosphorylation sites. Bub3 binding, conserved motif I and the TPR domain of Bub1 did not significantly contribute to Bub1 kinase activity, as measured by T589 and S679 phosphorylation (Fig. 2b,c). Kinetochore recruitment is therefore not required for Bub1 activation but serves to focus Bub1 kinase activity to kinetochores.

We were also intrigued by the recent suggestion that Bub1 is a constitutively active kinase based on the persistent phosphorylation of the P+1 autophosphorylation site S969 in G1 (ref. 19). To definitively test this, we verified Bub1 autophosphorylation at S679 (Fig. 2d) as well as H2A-T120 (Fig. 2e) in extracts from thymidine- and nocodazole-arrested cells. We find that neither Bub1-S679 nor H2A-T120 (in agreement with previous results^14^) was apparently phosphorylated in interphase extracts, although a clear signal was detected in extracts from mitotic cells, suggesting that Bub1 was not generally active during interphase. Nevertheless, we considered the possibility that the constitutive phosphorylation of S969 may reflect partial Bub1 activity, as has been previously suggested^19^. To test whether Bub1 may be further activated during interphase, we expressed 3 × MYC and *Lac* repressor (*LacI*)-fused Bub1 WT and Bub1 KD in cells stably expressing a 256 copy array of the *lac* operator sequence (*LacO*) in an arm of chromosome 1 (ref. 32) in an effort to artificially increase the localized concentration of Bub1. In interphase cells, LacI-tagged Bub1 WT and KD efficiently localized to the *LacO* array as indicated by anti-MYC immunofluorescence. In lacI-Bub1-WT- but not LacI-Bub1-KD-expressing cells or control cells, a clear overlapping signal was detected for H2A-pT120 and Sgo1 (Fig. 2f,g). Thus, increasing the local concentration of Bub1 is sufficient to induce its activation, even in the absence of kinetochores in interphase. This is in agreement with our data above showing that Bub1 activity is not dependent on Bub3 binding (Fig. 2b,c). Collectively, our results demonstrate that Bub1 phosphorylation at T589 and S679 occurs *in vivo* and establish that these are indeed autophosphorylation sites. Moreover, our data confirm and extend earlier observations demonstrating that Bub1 activation is primed already in interphase. We show that under normal circumstances, Bub1 is not sufficiently active in interphase but can be efficiently activated by increasing the local concentration.

### Bub1 T589 autophosphorylation regulates mitotic progression

We next focused on the role of T589 autophosphorylation, as this site is highly evolutionarily conserved (Fig. 2a). We generated stable isogenic HeLa lines expressing a single copy of triple MYC and green fluorescence protein (GFP)-tagged Bub1 WT, KD and T589A^12^ (Supplementary Fig. S2a,b). In *in-vitro* kinase assays, Bub1-T589A supported efficient H2A-T120 phosphorylation and mitotic arrest in the presence of nocodazole or taxol, suggesting that T589 phosphorylation is not implicated in regulation of the kinase activity or the SAC function of Bub1 (Supplementary Fig. 2c,d).

**Figure 3:**
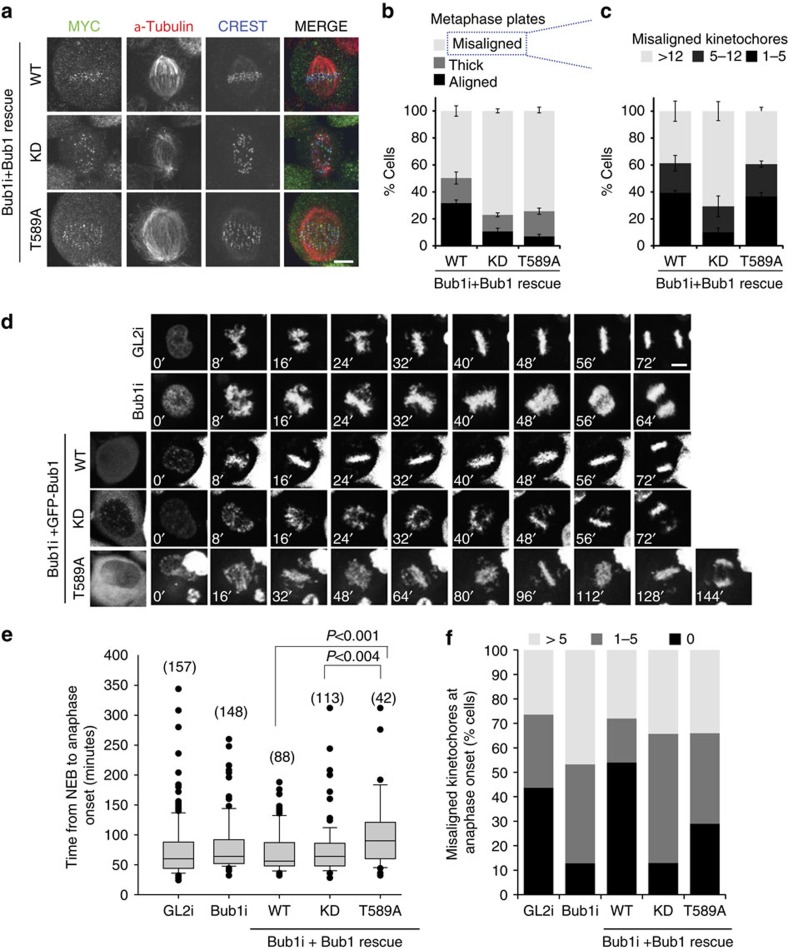
Loss of Bub1 phosphorylation at T589 causes chromosome congression defects. (**a**) Stable cell lines expressing Bub1-WT, Bub1-KD and Bub1-T589A were generated. See Supplementary Fig. 2 for characterization of the cell lines. Cells depleted of endogenous Bub1 were synchronized in mitosis as in Fig. 2a and then arrested for a further 2 h in MG132 before fixation and staining with anti-MYC (green), anti-α-tubulin (red) and anti-CREST (blue). (**b**) Quantitation of metaphase alignment from **a** was determined as described under Methods. A minimum of 100 cells were considered per condition in each of three replicates. Bars represent s.e. (**c**) The number of kinetochores from the ‘misaligned' category in **b** in the various Bub1-expressing lines. (**d**) Stills of the live-cell imaging of the cells lines and treatments indicated. Movies for Bub1-WT, KD and T589A-expressing cells are shown in Supplementary Movies 1–3, respectively. (**e**) Quantification of the mitotic timing of the experiment in **d**. The number of cells scored is indicated in parentheses. Significance is measured by *t*-test (two-tailed). (**f**) Quantification of lagging kinetochores at anaphase observed in **d**. The number of cells scored per condition is indicated in **e**. Scale bar, 10 μM.

We sought to test whether Bub1 autophosphorylation at T589 contributes to chromosome congression, which requires Bub1 kinase activity^12^,^29^,^33^. Stable Bub1 lines were depleted of endogenous Bub1 and were treated with MG132 to monitor congression. Whereas ∼50% of Bub1-WT-expressing cells aligned metaphase plates, only 23% of Bub1-KD and 26% of Bub1-T589A cells aligned at metaphase (Fig. 3a,b). The misalignment observed in cells expressing Bub1-KD was more severe than that observed in Bub1-T589A cells (71% in Bub1-KD cells versus 39% in Bub1-T589A cells with >12 misaligned kinetochores, Fig. 3c), indicating that T589 is not the only Bub1 substrate to contribute to chromosome alignment. We also assessed mitotic defects and progression in the Bub1 cell lines by live-cell imaging of progression through an unperturbed mitosis in cells co-expressing mRFP-Histone H2B, to permit chromosome visualization. We found no significant difference in the duration of mitosis between control (GL2 siRNA), Bub1-depleted cells and cells depleted of endogenous Bub1 but rescued with Bub1-WT and Bub1-KD, in agreement with previous reports^12^. Strikingly, cells expressing Bub1-T589A consistently required more time to complete mitosis, averaging 102 min between nuclear envelope breakdown (NEBD) and anaphase, whereas cells expressing WT and KD Bub1 required on average 71 and 75 min, respectively (Fig. 3d,e and Supplementary Movies S1–S3). In agreement with our observations in fixed samples, chromosome attachment defects were less pronounced in Bub1-T589A-expressing cells than in Bub1-KD cells (Fig. 3f). Our data demonstrate that Bub1 autophosphorylation at T589 contributes to proper chromosome congression and mutation of this residue causes a transient delay in mitosis.

### Bub1 autophosphorylation restricts H2A-pT120 to centromeres

**Figure 4:**
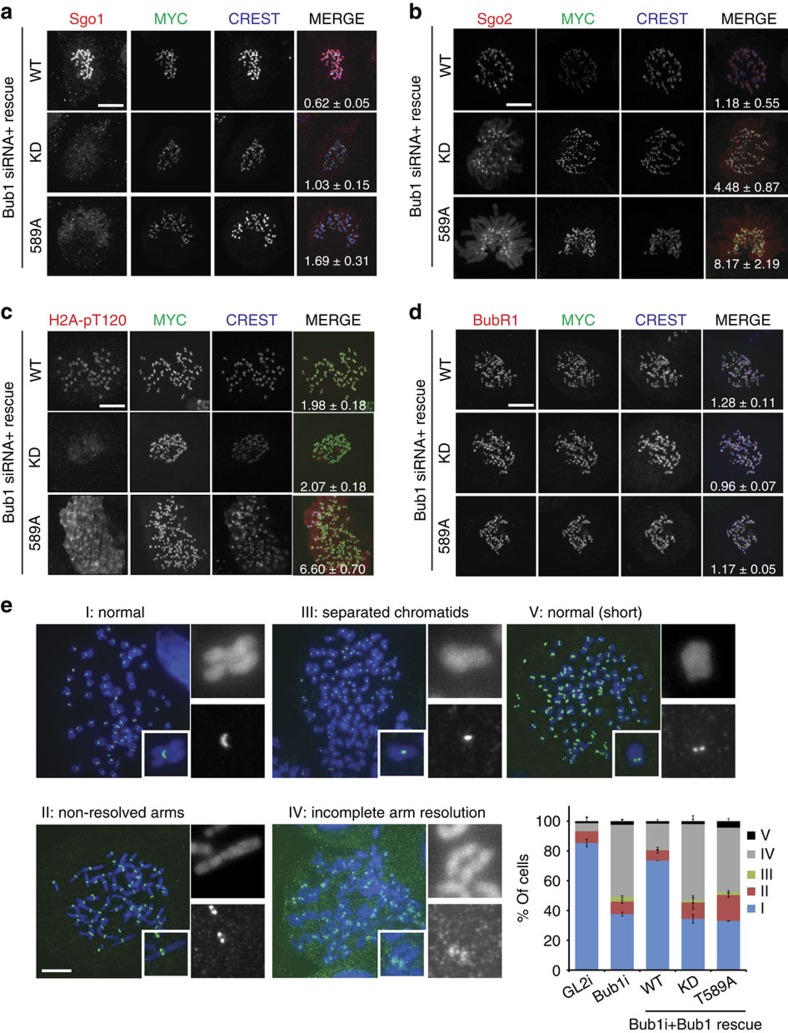
Uniform H2A-T120 phosphorylation results in ectopic Sgo recruitment and impaired sister chromatid resolution in cells expressing Bub1-T589A. (**a**–**d**) Mitotic Bub1-WT, KD and T589A depleted of endogenous Bub1 were fixed and stained with anti-CREST (blue) and anti-MYC (green), and (**a**) Sgo1, (**b**) Sgo2, (**c**) H2A-pT120 and (**d**) BubR1 (all in red). Quantification of immunofluorescence intensity specifically at the chromosome arms (corrected total cell fluorescence)±s.e. of Sgo1, Sgo2 and H2A-pT120 is indicated in the respective merge panel. For BubR1, fluorescence intensity relative to the CREST signal±s.e. is shown. (**e**) Stable Bub1 cell lines were depleted of endogenous Bub1, arrested in mitosis using nocodazole and harvested for chromosome spreads before staining with Hoechst (blue) and anti-GFP (green). The different chromosomal conformations were quantified and indicated in the graph. Data represent the mean±s.e. of 4 independent experiments, with 58–105 cells scored per condition per experiment. Scale bar, 10 μM.

The delay in mitotic progression in Bub1-T589A-expressing cells was somewhat surprising, considering that the more severe KD mutant exhibited normal timing. We reasoned that the effect of the T589A mutation on mitotic timing may be masked in the Bub1-KD, in which all Bub1 phosphorylation and activity are lost. To address this possibility, we sought to determine the effect of the T589A mutant on kinase-dependent Bub1 signalling. The H2A-pT120 centromeric mark generated by Bub1 recruits Sgo1 and Sgo2 to promote chromosome biorientation and proper chromosome segregation^14^; lack of Bub1 protein or Bub1 kinase activity has been reported to cause the spread of Sgo1 along the entire length of the chromosome^15^,^34^,^35^. In agreement with these observations, we find that Sgo1 is mislocalized to chromosome arms in cells expressing Bub1-KD, whereas Sgo1 is primarily localized to the centromere in cells expressing Bub1-WT (ref. 34 and Fig. 4a). Similar to Bub1-KD, expression of Bub1-T589A led to relocalization of Sgo1 to chromosome arms and the Sgo1 signal was more intense than that detected in Bub1-KD cells, an observation that was confirmed by corrected total cell fluorescence measurements directly on the chromosome arms (Fig. 4a and quantification within). Similarly, Sgo2 signal was detected at chromosome arms in cells expressing Bub1-KD, whereas it localized as expected to the centromere in Bub1-WT cells (Fig. 4b). Similar to Sgo1, expression of Bub1-T589A led to relocalization of Sgo2 to the chromosome arms (Fig. 4b), at levels considerably higher than seen in Bub1-KD-expressing cells. Nevertheless, a significant signal for Sgo2 could be clearly detected at the kinetochore, indicating that unlike Sgo1 a pool of Sgo2 remained insensitive to Bub1-KD and Bub1-T589A. We next examined the H2A-T120 phosphorylation under the same conditions. In cells expressing Bub1-WT, H2A-pT120 was clearly localized to the centromere but lost in Bub1-KD-expressing cells, as expected. Expression of Bub1-T589A, surprisingly, resulted in H2A-T120 phosphorylation along the entire length of the chromosome (Fig. 4c). Quantification of the H2A-pT120 signal specifically at chromosome arms revealed a significant increase in cells expressing this mutant compared with the essentially background staining-observed Bub1-WT- and Bub1-KD-expressing cells (Fig. 4c). To test whether the scaffolding function of Bub1 is altered by the loss of T589 phosphorylation, we verified the localization of BubR1. We found that at least steady-state levels of BubR1 are unchanged between cells expressing Bub1-WT, KD or T589A (Fig. 4d). Similarly, recent reports have concluded that Bub1 overexpression, which leads to H2A-pT120 spread to chromosome arms, did not alter the strength of the SAC or the recruitment of mitotic regulators^29^. Collectively, our data indicate that T589 autophosphorylation limits H2A-pT120 and hence Sgo to centromeres. The extended mitosis observed in Bub1-T589A cells may thus be a result of the longer time required to remove the ectopic cohesion resulting from unchecked H2A phosphorylation.

Sgo1 translocation to the chromosome arms after Bub1 inactivation induces persistent cohesion along mitotic chromosomes^15^. We therefore tested whether Bub1-T589A expression also resulted in ectopic cohesion using chromosome spreads. In control GL2-treated cells (85%) and rescued cells expressing Bub1-WT (74%), sister chromatids were predominantly X-shaped with only the centromere connection apparently maintained (Fig. 4e). As expected, cells depleted of Bub1 or depleted of Bub1 and rescued with Bub1-KD showed a significant increase in the proportion of cells with poor resolution of sister chromatids along the entire chromosome length (57% and 62%, respectively). Similarly, and in agreement with the mislocalization of Sgo proteins, cells expressing Bub1-T589A (61%) mostly displayed incomplete resolution along the length of chromosomes, presumably owing to unscheduled protection of cohesion caused by the spread of Sgo along the entire chromosome length. Together, these results suggest that in addition to H2A-T120 phosphorylation itself, Bub1 autophosphorylation at T589 is required to restrict H2A-T120 phosphorylation to the centromere, thereby confining Sgo and cohesion protection to this region.

### Bub1-KD and -T589A display increased cytoplasmic residency

**Figure 5:**
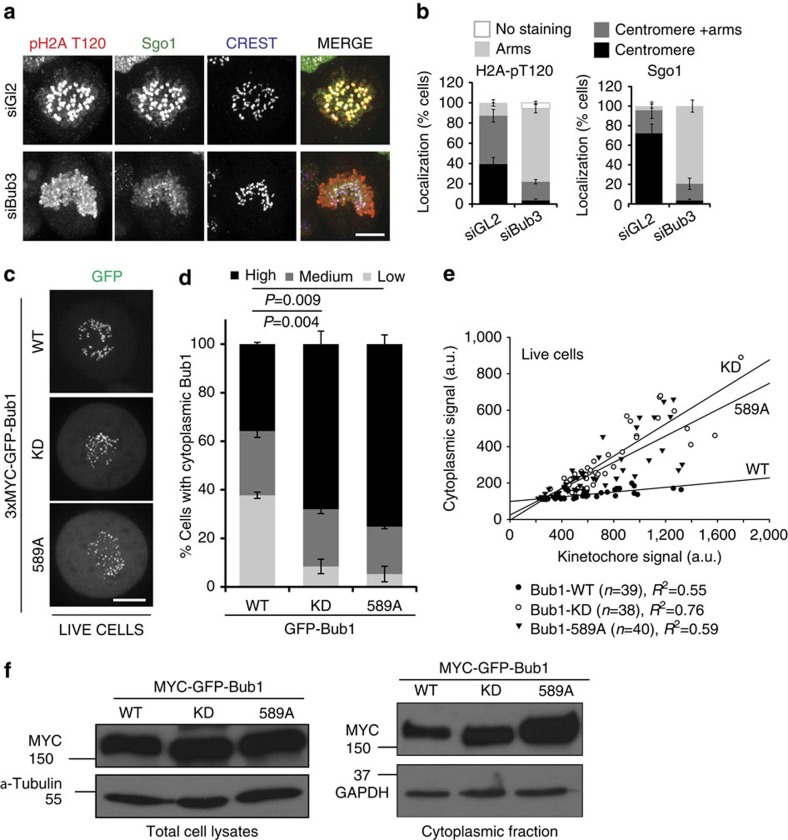
Bub1-KD and Bub1-T589A display increased residency in the cytosol. (**a**) Mitotic control (siGL2) and Bub3-depleted (siBub3) cells were fixed and stained with anti-H2A-pT120 (red), anti-Sgo1 (green) and anti-CREST (blue). (**b**) Quantification of the localization of H2A-pT120 and Sgo1 signals. Data represent the mean±s.e. of three independent experiments. Eighty to 300 cells were scored per condition per experiment. (**c**) Images and (**d**) quantification (normalized average pixel intensity); low (1–1.2), medium (>1.2 to ≤1.3) and high (>1.3) of 3 × MYC-GFP-Bub1 signal and localization in live cells synchronized in mitosis by a thymidine release. Data represent the mean±s.e. of 3 independent experiments, with 58–61 cells measured per condition. Significance was measured for the high group by one-way analysis of variance (ANOVA) and pairwise *t*-test (Holm–Sidak). (**e**) Scatter plot of the cytoplasm versus kinetochore GFP levels of individual cells from each of the stable cell lines. The number of cells, *R*^2^ (measure of the goodness-of-fit) and significance (one-way ANOVA) are indicated. (**f**) Western blottings showing levels of the 3 × MYC-GFP-Bub1 proteins in the stable cell lines in whole cell extracts (left) and in cytoplasmic extracts (right). Scale bar, 10 μM.

Loss of localized H2A-T120 phosphorylation in Bub1-T589A cells was also seen in KNL1-depleted cells^19^ and suggested that kinetochore targeting of Bub1 enriches H2A-T120 phosphorylation at centromeres^14^. To independently verify these observations, we depleted Bub3, the constitutive binding partner of Bub1 that is strictly required for Bub1 kinetochore binding through interaction with KNL1 (refs 36, 37, 38) reviewed in ref. 8). Bub3 depletion results in efficient relocalization of Bub1 to the cytoplasm, as expected (ref. 39 and data no shown). Concomitant to this loss, we observed a massive spread of H2A-T120 phosphorylation along chromosome arms and a corresponding recruitment of Sgo1 (Fig. 5a,b). These results are in strong agreement with the observation that Bub3 binding is not required for Bub1 activity *per se*, but rather to focus Bub1 activity to kinetochores (Fig. 2b,c), and argue that loss of Bub3–Bub1 concentration at the kinetochore results in ectopic H2A-T120 phosphorylation and Sgo1 recruitment^19^, probably through the activity of cytoplasmic Bub1.

The parallels in the phenotype observed in Bub3-depleted cells and Bub1-T589A cells were surprising, considering that Bub1-T589A localized efficiently to the kinetochore, as measured by indirect immunofluorescence, and exhibited normal activity towards H2A *in vitro* (Supplementary Fig. 2B,C). To determine directly whether Bub1-T589A resided in the cytoplasm and to avoid potential artefacts from fixation, we monitored the localization of enhanced GFP-tagged Bub1 in our isogenic cell lines in living mitotic cells. We measured the cytoplasmic expression using three independent approaches. First, we monitored Bub1 expression in undisrupted prometaphase cells. Approximately 38% of the cells expressing Bub1-WT showed low or undetectable levels of GFP signal in the cytoplasm, in agreement with Bub1 residency being primarily at the kinetochore. Surprisingly, we found that in Bub1-KD- and Bub1-T589A-expressing cells, this percentage was much lower with ∼8% and 5% of cells exhibiting low cytoplasmic GFP levels, respectively. Conversely, proportionally more Bub1-KD and T589A cells displayed high GFP signal in the cytoplasm when compared with Bub1-WT-expressing cells (Fig. 5c,d). As an alternative approach, we plotted the cytoplasmic versus kinetochore GFP-Bub1 signal of individual cells in a random population of mitotic cells from each of the cell lines. Linear regression analysis indicated that Bub1-KD- and Bub1-589A-expressing cells tended to display higher cytoplasmic versus kinetochore ratios than Bub1-WT (Fig. 5e). Although no significant difference was observed between Bub1-KD and Bub1-T589A cells (*P*=0.36), the cytoplasmic:kinetochore GFP ratios in these cells were found to be significantly higher than the cells expressing Bub1-WT (*P*<0.001, one-way analysis of variance (ANOVA); Fig. 5e). Finally, we tested the overall expression in these Bub1 cell lines, as well as the proportion of the protein that was found in the cytoplasmic compartment after fractionation. Western blotting indicated that Bub1-WT, KD and T589A are expressed at similar overall levels (Fig. 5f, left panel). However, when taking just the cytoplasmic fraction in consideration, both Bub1-KD and Bub1-T589A displayed higher cytoplasmic levels (Fig. 5f), in agreement with our aforementioned results. Taken together, our observations suggest that although Bub1-WT, -KD and -T589A cell lines express similar overall levels of Bub1, Bub1-KD and Bub1-T589A exhibit higher cytoplasmic occupancy than Bub1-WT.

### Kinetochore-tethered Bub1-T589A refocuses H2A-pT120 and Sgo

**Figure 6:**
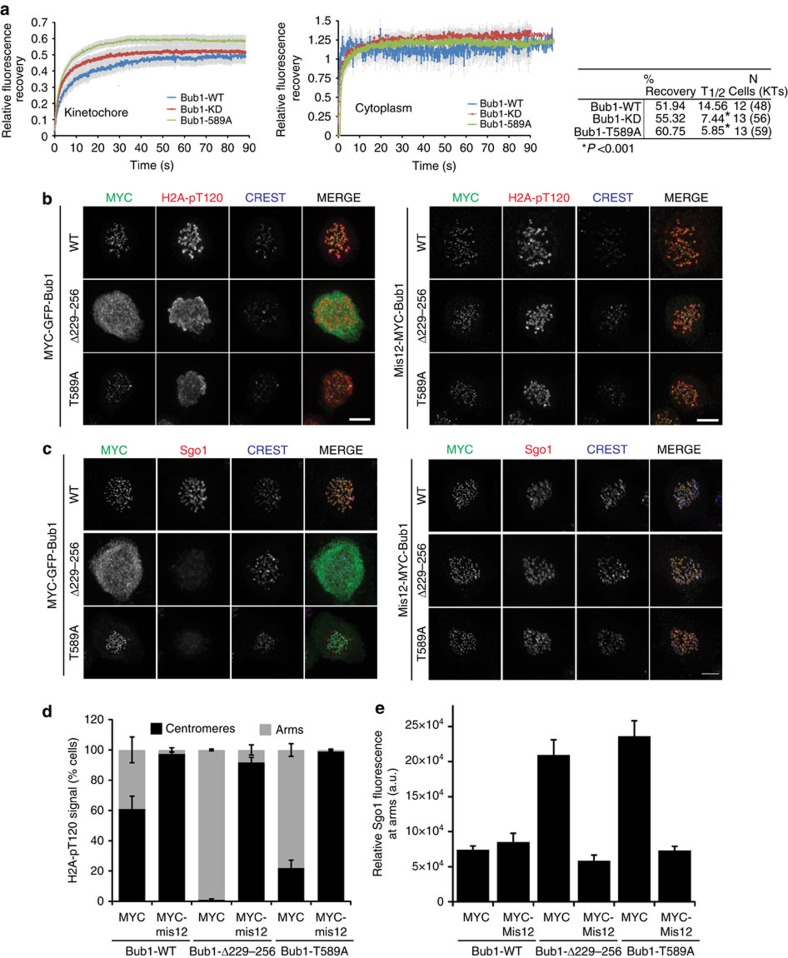
Bub1-KD and Bub1-T589A display aberrant kinetochore shuttling dynamics. (**a**) Recovery analysis of 3 × MYC-Bub1 WT, KD and T589A after FRAP analysis at the kinetochore (left) and cytoplasm (right). Recovery parameters for the kinetochore population are shown in the table on the right. Statistical analysis was performed by analysis of variance. (**b**) Mitotic cells expressing MYC-tagged (left panels) or MYC-Mis12-tagged (right panels) Bub1-WT, T589A and Δ229–256 were fixed and stained with anti-H2A-pT120 (red), anti-MYC (green) and anti-CREST, respectively. (**c**) Cells treated as in **b** but were stained with Sgo1 (red). (**d**,**e**) Quantification of the phenotypes observed in **b** and **c**, respectively. For **d**, the data is the mean±s.e. of 3 independent experiments, with 80–100 cells scored per condition per experiment.

As Bub1-T589A appeared to localize normally to kinetochores (Supplementary Fig. 2a,b), we examined whether an increase in exchange at kinetochores caused aberrant cytoplasmic presence by measuring FRAP. After photobleaching at kinetochores, Bub1-WT recovered to ∼52% (Fig. 6a) in agreement with previous observations in PtK_2_ cells^40^ and fission yeast^11^, with a half-life of ∼15 s. Recovery of Bub1-KD and Bub1-T589A increased marginally to 55% and 61%, respectively. Recovery occurred with significantly faster kinetics with half-life measurements of 7.44 s for Bub1-KD and 5.85 s for Bub1-T589A (*P*<0.001, one-way ANOVA). In contrast, we found no major difference in cytoplasmic diffusion (Fig. 6a). This data suggests that Bub1 kinase activity and, in particular autophosphorylation at T589, restricts the kinetics as well as the fraction of Bub1 exchanged between kinetochores and the cytoplasm.

We next reasoned that if increased Bub1-T589A kinetochore turnover was indeed causing uniform H2A-pT120 and Sgo1 recruitment to chromatin, then stable tethering of Bub1-T589A to the kinetochore would refocus H2A-T120 phosphorylation. To test this idea, we expressed MYC-tagged Bub1 WT, the Bub3-binding mutant Δ259–276 and T589A or their Mis12 chimeras to stably incorporate Bub1 at kinetochores. In the majority of Bub1-WT-expressing cells, H2A-pT120 was centromeric and this proportion was further increased in cells expressing the Mis12-Bub1WT, in accordance with the stable docking of Mis12 at kinetochores (Fig. 6b,c and ref. 41). As expected, expression of Bub1-Δ259–276 and Bub1-T589A caused a significant increase in the proportion of cells with H2A-pT120 staining at chromosome arms. Strikingly, in cells expressing Mis12-Bub1-T589A and Mis12-Bub1-Δ259–276, the H2A-pT120 signals concentrated at kinetochores in over 90% of the cells, effectively rescuing the aberrant H2A-T120 arm phosphorylation seen in these mutants (Fig. 6b,d). In line with the role of H2A-pT120 as a major receptor for Sgo1 at kinetochores, Mis12-Bub1-T589A efficiently targeted Sgo1 to kinetochores (Fig. 6c,e). Thus, ectopic phosphorylation of H2A-T120 and Sgo1 recruitment resulting from Bub1-T589A (which inappropriately shuttles between the kinetochore and cytoplasm) and Bub1-Δ259–276 (which does not localize to the kinetochore at all) can be effectively rescued by artificial tethering of Bub1 to kinetochores.

## Discussion

Many protein kinases undergo autophosphorylation in the course of catalysis. In the activation segment, a conserved structural element within the kinase domain, phosphorylation stabilizes the catalytically active state of many eukaryotic protein kinases^42^ and often occurs through autocatalysis. Although SAC kinases are known to be highly autophosphorylated, the current picture of the function of this autophosphorylation is far from being complete.

Here we show that Bub1 becomes highly autophosphorylated during mitosis at a number of conserved sites outside the activation segment including T589 and S679. This activity requires the kinase extension domain, but not the TPR domain, kinetochore recruitment or Bub3-binding. Recruitment to the kinetochore by Bub3 instead serves to concentrate Bub1 activity at kinetochores. Although it is now clearly established that bulk kinetochore recruitment of Bub1-Bub3 occurs through binding to KNL1 after Mps1 phosphorylation of MELT sequences^8^,^36^,^37^,^38^,^43^,^44^,^45^,^46^, autophosphorylation at the highly conserved T589 is required for proper Bub1 kinetochore–cytoplasm shuttling, which is in turn required for accurate mitotic progression by ensuring localized H2A-T120 phosphorylation and Sgo recruitment. Kinetochore tethering of either Bub1-T589A or the Bub3-binding mutant Bub1-Δ229–256 via Mis12 refocuses H2A-T120 phosphorylation and Sgo1 to the centromere. Our study reveals an additional regulatory layer controlling Bub1 localization.

Considerable evidence from the literature supports this model of Bub1 function. First, all conditions in which proper Bub1 kinetochore targeting is impaired result in the spread of the H2A-pT120 signal and/or Sgo1 displacement along chromosome arms. Our data here show that depletion of Bub3 or loss of the Bub1–Bub3 interaction result in unchecked H2A-T120 phosphorylation and Sgo recruitment. Similarly, depletion of KNL-1 or ectopic localization of the Bub1 kinase domain to chromosome arms led to uniform H2A-T120 phosphorylation on chromatin^14^,^19^. In fission yeast, expression of Bub1 lacking the amino-terminal kinetochore-targeting region elevated H2A-S121 phosphorylation along the entire chromosome length^14^. Interestingly, depletion or inhibition of Aurora B kinase also affects Sgo1 localization by causing its redistribution to chromosome arms during mitosis and meiosis^47^,^48^,^49^,^50^,^51^. This may be at least in part due to the loss of Bub1 kinetochore targeting in the absence of Aurora B activity^35^,^52^,^53^. In addition to our observations with Bub1, both Aurora B and Mps1 have been reported to contribute to their own localization through autophosphorylation. In *Saccharomyces cerevisiae*, Ipl1 (the budding yeast Aurora B orthologue) and Cdk1 phosphorylation of a number of consensus sites in Sli15/INCENP restricted premature chromosomal passenger complex localization to the spindle^54^,^55^ but left Ipl1 activity unchanged. Similarly in vertebrates, Aurora B activity is strictly required for proper loading of the complex at centromeres^56^ and the central spindle^57^,^58^. Mps1 exchange is also dependent on its own kinase activity^59^. More inactive than active Mps1 can be detected at kinetochores by immunofluorescence and FRAP analysis indicated faster recovery (1.5-fold) of inactive Mps1 at the kinetochore^59^. More recently, it has been suggested that Mps1 autophosphorylation at a number of N-terminal sites outside the kinase domain reduces the affinity of Mps1 to kinetochores and may thus underlie this exchange^60^. The ability to regulate their own localization may therefore be a hallmark of the structurally and functionally diverse kinases that orchestrate mitosis.

**Figure 7:**
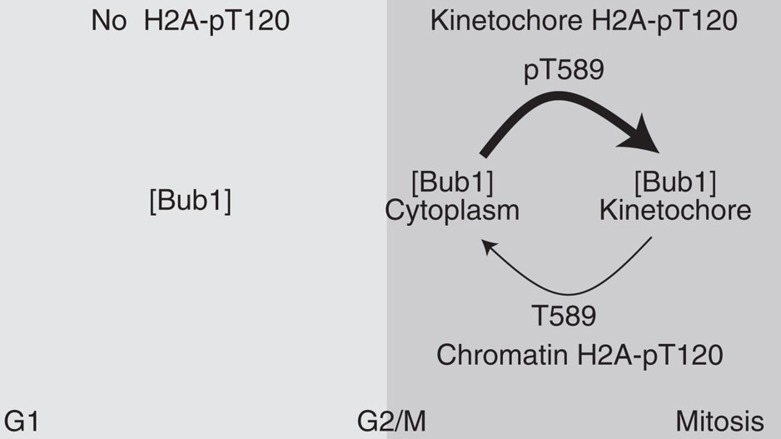
Model of Bub1 activation and autoregulation. See text for full discussion of the model.

Crystal structures suggest that Bub1 may be a constitutively active kinase^19^,^61^ and Bub1 is autophosphorylated at S969 in the activation segment throughout the cell cycle. However, it is clear that not all Bub1 phosphorylation events occur during interphase (Fig. 2d,e and ref. 14). To reconcile these results we propose that a critical concentration of Bub1 must be reached before activation (see model in Fig. 7). In interphase, the concentration of Bub1 is low and thus the kinase remains effectively inactive. In agreement with this, Bub1 is degraded at the end of mitosis in an APC/Cdh1-dependent manner and its levels drop rapidly on entry into G1 (refs 24, 62). Indeed, increased local concentration of Bub1 is sufficient for activation and H2A-T120 phosphorylation, and Sgo1 recruitment during interphase (Fig. 2f,g). At G2/M, Bub1 protein expression increases and the critical threshold would be achieved resulting in Bub1 activation. In support of this idea, H2A-S121 is phosphorylated and Sgo2 is recruited along the entire chromosome length in G2 in a Bub1-dependent manner in fission yeast^14^. On mitotic entry and the establishment of kinetochores, this activity becomes concentrated in the vicinity of its targets through Bub3–Bub1 binding to KNL1. As cytoplasmic Bub1 remains capable of phosphorylating H2A-T120 during mitosis albeit ectopically (Figs 5a and 6b, and ref. 14), kinetochore targeting spatially restricts, rather than activates, Bub1 (ref. 19). T589 autophosphorylation further restricts Bub1 shuttling between the cytoplasm and kinetochore. This is necessary because the increased cellular concentration of Bub1 during mitosis is sufficient to activate it and induce otherwise indiscriminate H2A-T120 phosphorylation and Sgo recruitment in these mutants. The effect of this dynamic exchange in Bub1-KD cells is masked due to the lack of H2A-T120 phosphorylation altogether. Loss of this autoregulatory phosphorylation results in ectopic cohesion protection, owing to mislocalized Sgo and a significant prolongation of mitosis, perhaps reflecting the additional time required to remove Sgo and cohesion from along chromosome arms. In support of this notion, a similar transient delay in exit was reported in cells depleted of WAPL, a protein required for the timely removal of cohesion in prophase^50^. Thus, the role of Bub1 in Sgo localization and cohesion protection is twofold: first, Bub1 directly phosphorylates H2A-T120 to mediate Sgo recruitment and, second, through feedback autophosphorylation at T589, Bub1 ensures that H2A-pT120 and Sgo are restricted to kinetochores. Constitutive autophosphorylation of S969 in the P+1 loop of Bub1 (which occurs by intramolecular phosphorylation and is independent of Bub1 concentration^19^) may function as a priming event to ensure rapid and efficient H2A-T120 (and T589) phosphorylation on mitotic entry. Activation of Bub1 may thus not be switch-like and may involve intermediate states that exhibit varying degrees of activity^61^.

The H2A-pT120-Sgo1 pathway serves as an adaptor to facilitate Aurora B inner centromeric accumulation^63^,^64^,^65^. We therefore checked both localization and activation of Aurora B. We found that neither Aurora B protein levels nor Aurora B activity, as measured by autophosphorylation on T232 or phosphorylation of the canonical substrate CENPA-S7 was appreciably different between Bub1-WT and Bub1-T589A cells, although all three signals were diminished in Bub-KD cells, as expected (Supplementary Fig. 3). Considering that depletion of both Sgo1 and Sgo2 is necessary to mislocalize Aurora B^63^, that we observed appreciable levels of Sgo2 at kinetochore and that we found no effect of Bub1 autophosphorylation on the Haspin-generated pH3T3 marker that recruits survivin to centromeres^65^, it is likely to be that sufficient Aurora B is recruited and activated at centromeres in the Bub1-T589A-expressing cells. Although we cannot rule out a minor effect on Aurora B activity that is beyond the resolution offered by the phosphospecific antibodies used in this study, the congression defects observed may be due to a reduction in centromeric Sgo2, which is required for mitotic centromere-associated kinesin recruitment^66^. We also found that steady-state BubR1 levels as measured by immunofluorescence (IF) are unchanged in the Bub1-T589A mutant. However, considering that BubR1 kinetochore binding occurs directly through Bub1 (ref. 67), it may well be that BubR1 kinetochore turnover (rather than bulk levels) is also altered in the Bub1 T589A mutant. Answering this question will require further investigation.

How T589 phosphorylation changes the dynamics of Bub1 shuttling remains unclear. One possibility is that phosphorylation may induce conformational changes that alter affinity of Bub1 to kinetochores. Binding of Bub3 as measured by immunoprecipiation revealed no difference between this mutant and Bub1-WT however, and any change caused by this mutation might be small and restricted to the shuttling pool of Bub1 (∼ 50%) and thus not easily detected by such steady-state assays. Alternatively, it is possible the pT589 motif forms a docking site for a protein interaction motif, which would allow for the dynamic exchange of Bub1 between the cytoplasm and kinetochores. For example, 14–3–3 binding to pThreonine motifs has been shown to control the nucleocytoplasmic exchange of a number of proteins^68^. Whether a similar mechanism regulates Bub1 exchange remains to be explored.

## Methods

### Cell culture and transfection

All cell lines were grown at 37 °C with 5% CO_2_ in DMEM (Hyclone) supplemented with 10% fetal bovine serum or supplemented bovine growth serum (PAA). HeLa cells for the generation of stable isogenic cell lines were a generous gift of Patrick Meraldi^12^. Stable lines were generated by electroporation essentially (Flp-In system, Life Technologies) and were selected for and grown in the presence of hygromycin (300 μg ml^−1^). U20S cells expressing a 256 copy array of the Tet operator sequences were a kind gift of David Spector (CSHL^32^) and were maintained in the presence of 100 μg ml^−1^ of hygromycin. Drug treatments were performed at the following concentrations and durations, unless otherwise indicated: thymidine (Acros Organics, 2 mM for 16 h), MG132 (Calbiochem, 20 μM for 2 h) and nocodazole (Sigma, as indicated, 16 h). Transient plasmid transfections (Figs 2 and 6) were performed with jetPRIME or TransIT (Polyplus) according to the manufacturer's instructions. Protein depletion was performed with DsiRNAs (IDT), unless otherwise indicated, using either oligofectamine (Invitrogen) or INTERFERin (Polyplus) and analysed at 48–72 h after transfection. The Bub1 siRNA pool targeting the 3′-UTR region has been previously reported^31^. The DsiRNA equivalent was generated and correspond to the following sequences: Bub1–3′UTR-1: 5′-UCCCAUGGAAUAUUUCCAUGUAAAA-3′; Bub1–3′UTR-2: 5′-UCACACUGUAAAUAUGAAUCUGCTC-3′; Bub1–3′UTR-3: 5′-AAAAAACAGGUUUAAAGUGAGCAGAUU-3′; and Bub1–3′UTR-4: 5′-UUUAAGGACUGUCUAUAUCCAAAUUUU-3′. The Bub3-siRNAs used target the following sequences: siBub3–1: 5′-UGACAGAUGCAGAAACAAAdTdT-3′and DsiBub3–2: 5′-AGGGUUAUGUAUUAAGCUCUAUUGA-3′.

### Chromosome spreads

The Bub1 stable cell lines were split into six-well plates and transfected with Bub1 3′-UTR DsiRNA. At 36 h post transfection, cells are synchronized with 330 nM nocodazole overnight. Mitotic cells were shaken off, washed three times in PBS and incubated for 15 min at 37 °C with rotation in 55 mM KCl to swell the cells. Twenty thousand cells were subsequently resuspended in 200 μl of 55 mM KCl, 0.1% Tween 20 and chromosomes were spread onto coverslips by centrifugation at 500*g* in a cytospin with slow acceleration and deceleration. Cells attached on slide were fixed in PTEMF for 10 min as before^69^. Spread chromosomes were then stained as indicated in the figure legends.

### Cloning and mutagenesis

Bub1 WT was cloned into the pcDNA5/FRT/TO expression vector modified to include an N-terminal triple MYC tag (HindIII–Kpn1) followed directly in-frame by EYFP (KpnI–BamHI), generated by PCR amplification from pEGFP-N1 (Clontech). For artificial kinetochore targeting of Bub1-T589A and Bub1-Δ229–256, the enhanced GFP sequence was substituted for hMis12. For construction of the LacI-expressing plasmid, *LacI* was amplified from pSV2-YFP-LacI (David Spector) and subcloned into the KpnI site of pcDNA3.1–3xMYC^70^. Bub1-WT and Bub1-KD were subsequently cloned into this vector using BamHI and XhoI. Bub1 KD (D946A), T589A and S679A were generated by QuickChange site-directed muagnenesis. Bub1-Δ150 lacking the first 150 amino acids was generated by PCR. Bub1 plasmids for mutants Δ229–256, Δ740–766 and Δ458–476 were a gift from Patrick Meraldi^12^.

### Immunofluorescence and antibodies

Cells were grown on cover slips and were arrested in mitosis either by nocodazole (330 nM) or by 10–12 h release from thymidine block. Fixation was performed by incubation with PTEMF (0.2% Triton X-100, 20 mM PIPES pH 6.8, 1 mM MgCl_2_, 10 mM EGTA and 4% formaldehype)^69^ before blocking in 3% BSA in PBS-T. Coverslips were incubated with primary and secondary antibodies for 1 h at room temperature, except for CENP-A pS7, which was incubated at 4 °C overnight. Rabbit polyclonal phosphospecific antibodies against T589 and S679 were generated against phosphopeptides CIRCNKpTLAPS and CLLRLpSQPAAG, respectively. Antibodies were affinity purified with the phosphopeptide for all experiments shown here and used at 1 μg ml^−1^. Other antibodies were used at 1 μg ml^−1^, unless otherwise indicated, as follows: anti-MYC (9E10, Thermo Scientific), anti-Bub1 (ref. 70), anti-GFP (11814460001, Roche), anti-SgoL1 (H00151648-M01, Abnova), anti-H2ApT120 (61195, Active Motif and a generous gift of Y. Watanabe), anti-α-tubulin (DM1A, Santa Cruz), anti-Sgo2 (kind gift of Tim Yen), anti-GAPDH (used at 1:2,000, NB300–221, Novusbio), CENP-A pS7(used at 1:100, 2187, Cell Signaling Technology), anti-Aurora B (611082, BD Transduction Laboratories), anti-Aurora pT232 (600–401–677, Rockland), anti-histone H2A (07–146, Millipore) and CREST anti-centromere serum (HCT-0100, Immunovision). Dylight series secondary antibodies (Thermo) were used for immunofluorescence (1:1,000) and horseradish peroxidase-coupled secondary antibodies (Jackson ImmunoResearch) were used for western blotting (1:10,000).

### Protein detection and fractionation

For immunoblotting and immunoprecipitations, cells were lysed with RIPA buffer containing 150 mM Tris-HCL pH 7.5, 150 mM NaCl, 10 mM NaF, 1% NP-40, 0.1% Na deoxycholate and a protease and phosphatase inhibitor cocktail that included 20 mM B-glycerophosphate, 0.1 mM sodium vanadate, 10 mM sodium pyrophosphate, 1 μg ml^−1^ leupeptin, 1 μg ml^−1^ aprotinin and 1 mM AEBSF. Cells were lysed on orbital shaker at 4 °C for at least 30 min; lysates were centrifuged at 14,000 r.p.m. for 15 min at 4 °C. The supernatant was collected and protein concentrations were measured using the BCA assay (Thermo Scientific). For isolation of the cytoplasmic and cytoskeletal fraction of Bub1, mitotic cells stably expressing Bub1-WT, KD or T589A were harvested by shake-off after thymidine release, washed twice in PBS and lysed for 10 min on ice in cytoskeletal buffer (0.5% Triton X-100, 100 mM PIPES pH 6.8, 100 mM NaCl, 1.5 mM MgCl2, 300 mM sucrose, protease inhibitor cocktail (1 μg ml^−1^ aprotinin, 1 μg ml^−1^ leupeptin, 1 mM AEBSF, 10 mM NaF) and 1 mM ATP). The lysate was spun down for 4 min at 3,200 r.p.m. and the resulting supernatant (S1) constituted the cytoplasmic fraction. The original, non-cropped blots for all western blottings in this study are shown in Supplementary Fig. 4

### Microscopy and FRAP

Cells were imaged by confocal microscopy on an inverted Olympus IX80 microscope equipped with a WaveFX-Borealin-SC Yokagawa spinning disc (Quorum Technologies) and an Orca Flash4.0 camera (Hamamatsu). Image acquisition was performed using Metamorph software (Molecular Devices). Optical sections were acquired with identical exposure times for each channel within an experiment and then projected into a single picture using ImageJ (rsb.info.nih.gov). Image processing was performed in Image J or Photoshop and images shown in the same figure have been identically scaled.

For FRAP experiments, the cells were grown in glass-bottom lab-tek chambered slides (Thermo Scientific). FRAP analysis was performed on Leica DMI600B equipped with a heated chamber (37 °C) and a Mosaic active illumination system (Spectral Applied Research), which allowed for simultaneous bleaching and acquisition, and an ImageEM (512 × 512) camera (Hamamatsu). The microscope and Mosaic were operated by Metamorph. The GFP-tagged Bub1 protein at both kinetochores and the cytoplasm was bleached using a 405-nm laser (diode 475 mW power at 100%) and excited at 491 nm (detection filters 536/40 nm). Individual kinetochores or cytoplasmic regions were bleached by a 400-ms laser pulse. Image acquisition (every 150 ms) began 15 frames before bleaching and continued for an additional 750 frames post bleaching. The bleached region in each case was a circular region of 15 pixel diameter and only kinetochores that remained visible within this region for the length of the experiment were included in the analysis. Quantification of fluorescence recovery was obtained using the FRAP profiler plugin of ImageJ, which accounts for correction of overall bleaching. Recovery rates for cytoplasmic and kinetochore Bub1 WT, KD and T589A were determined after fitting a single exponential curve (which showed the best fit) using the formula *F*(*t*)=*A*(1−*e*^−*τt*^), where *A*=fraction recovery. Half-time recovery was determined using the formula *T*_1/2_=ln0.5/−*τ*.

Live-cell imaging was performed on the above indicated microscopy system that is also equipped with a motorized stage (ASI) and an incubator with atmospheric CO_2_ heated to 37 °C. Bub1 stable cell lines were subjected to depletion of endogenous Bub1 for 48 h, then synchronized in mitosis after a further 16-h block with thymidine. Image acquisition was started 12 h after release. Only cells visibly expressing the GFP-tagged Bub1 were included in subsequent analysis.

### SILAC labelling and MS analysis

293T cells were cultured in heavy or light amino acid-containing medium for five generations before transfection with 3 × MYC-tagged Bub1-WT or Bub1-KD. Cells were incubated for 36 h, after which nocodazole was added for an additional 16 h. Cells were harvested, lysed in RIPA lysis buffer and 3 × MYC-tagged Bub1 WT or KD were immunoprecipitated with anti-MYC antibodies for 2 h. The immunoprecipitated Bub1 was washed 3 × with RIPA lysis buffer, 1 × with RIPA buffer including 300 mM NaCl and a final buffer exchange with kinase reaction buffer lacking ATP and MgCl_2_. The immunoprecipitates were then subjected to a cold *in-vitro* kinase assay (20 mM Tris pH 7.4, 10 mM EGTA, 100 mM sodium orthovanadate, 10 mM MgCl_2_, 4 mM MnCl_2_, 1 mM dithiothreitol, 5 mM NaF and 100 μM ATP) at 30 °C for 30 min. The reaction was stopped by the addition of SDS–PAGE sample buffer. The Bub1-WT and Bub1 KD immunoprecipitates were then mixed, resolved by SDS–PAGE and visualized by Coomassie brilliant blue staining. The band corresponding to the size of 3 × MYC-Bub1 was excised and processed for MS analysis^69^. In-gel digestion was performed using either 15 ng ml^−1^ of trypsin or was added in an enzyme/substrate ratio of 1:50 of each Lys-C, GluC and elastase.

### Nano liquid chromatography–MS/MS analysis

All peptide samples were separated by online reverse-phase nano liquid chromatography and analysed by electrospray tandem MS (MS/MS). Using a nanoACQUITY ultra-performance liquid chromatography system (Waters), samples were injected onto a 14-cm fused silica capillary column with an inner diameter of 75 μm and a tip of 8 μm (New Objective) packed in-house with 3-μm ReproSil-Pur C18-AQ (Dr Maisch GmbH). The LC setup was connected to an LTQ-Orbitrap MS (Thermo Fisher Scientific) equipped with a nanoelectrospray ion source (Proxeon Biosystems). Peptides were separated and eluted by a stepwise 180 min gradient of 0%−100% between buffer A (0.2% formic acid in water) and buffer B (0.2% formic acid in acetonitrile). Data-dependent acquisition was performed on the LTQ-Orbitrap using Xcalibur 2.0 software in the positive ion mode. Survey full-scan MS spectra (from *m/z* 300 to 2,000) were acquired in the FT-Orbitrap with a resolution of 60,000 at *m/z* 400. A maximum of five peptides were sequentially isolated for fragmentation in the linear ion trap using collision-induced dissociation. The Orbitrap lock mass feature was applied to improve mass accuracy. To improve phosphopeptide analysis, the multistage activation option in the software was enabled and the neutral loss species at 97.97, 48.99 or 32.66 *m/z* below the precursor ion were activated for 30 ms during fragmentation (pseudo-MS^3^).

### Data processing and analysis

Raw data files including SILAC quantitation were processed using the MaxQuant software suite (version 1.0.12.5). Generation of peak lists was performed with the following MaxQuant parameters: top 12 MS/MS peaks for 100 Da, 3 data points for centroid, Gaussian centroid determination and slice peaks at local minima. During the peak list generation, MaxQuant identified potential SILAC pairs based on mass differences of specified labelled amino acids, intensity correlation over elution time and so on. Mascot (version 2.2.0, Matrix Science) was used for peptide identifications. The initial precursor mass tolerance was set to ±7 p.p.m., whereas an accuracy of ±0.5 Da was used for MS/MS fragmentation spectra. Carbamidomethylation was set as fixed modification and methionine oxidation, protein N-terminal acetylation and phosphorylation (STY) were considered as variable modifications. Putative SILAC pairs were searched with their respective labelled amino acids as fixed modification, whereas peaks that were not assigned to any of the SILAC pairs were searched using R10 and K8 as variable modifications. Enzyme specificity was set to Trypsin/P, that is, allowing cleavage N-terminal to proline in the context of [KR]P. Up to two missed cleavages were allowed. The minimum required peptide length was set to six amino acids. Searches were performed against IPI human (version 3.48; 71,400 protein entries) that was concatenated with reverse database sequences (142,800 protein entries in total). Further, MaxQuant filtered Mascot results using additional parameters such as the number of labelled amino acids (maximum of three) in the identified peptide sequence and the measured mass accuracy as a function of intensity. As an additional quality measure to increase identification stringencies, we only accepted phosphorylation site identifications with Mascot scores of at least 12 or PTM scores of at least 30. Quantitation of SILAC pairs was performed with the following parameters: re-quantify; for protein quantification, discard unmodified counterpart peptides, except for oxidation and acetyl protein N-terminal; use razor and unique peptides; minimum ratio count of 1; minimum score 0; and minimum peptides 1. The initial maximum false discovery rates were set to 0.02 and 0.05 for peptides and proteins, respectively, and further reduced by Mascot score filtering as described above. False discovery rates were calculated as (number of hits in the reversed database/number of hits in the forward database) × 100%. Whenever the set of identified peptides in one protein was equal to or completely contained in the set of identified peptides of another protein, these two proteins were joined in a single protein group. In cases where the peptides have more than one phosphorylation site, some of these phosphorylation sites are identified as multiply phosphorylated peptides, whereas others are identified on multiple singly phosphorylated peptides.

### Phosphopeptide analysis

A summary of all quantifiable SILAC pairs identified on Bub1 from each of the three independent experiments is shown in Supplementary Data 1. It is worth noting that for experiment 2, in a certain number of cases, peptides corresponding to Bub1-KD were identified but lacked phosphorylation all together. In these cases, we marked the phosphopeptide:peptide ratio as 100%. Weblogo analysis was performed on the 15-mer peptides corresponding to the 20 autophosphorylation sites identified in this study together with H2A-T120 using the default settings.

### Quantification and statistical analysis

Unless otherwise stated, all experiments were performed in triplicate. Image quantification was performed using Image J. For quantification of signal intensities at kinetochores, the CREST/MYC signal was used to generate a binary mask to include kinetochore and centromere signals. Integrated signal intensity was measured in all relevant channels and intensities indicated are values relative to CREST or MYC. Unless otherwise indicated, a minimum of ten cells were quantified per condition for all experiments involving kinetochore quantification. For H2A-pT120 and Sgo1 signal intensity at chromosome arms, the following formula was used to measure the corrected total cellular fluorescence. CTCF=integrated density−(area of selected cell × mean fluorescence of background readings). For Fig. 2f,g, signal overlap was measured by drawing a 70 pixel line across the MYC-Bub1 signal and the intensity of the co-localized Sgo1 and histone H2A-pT120 was measured along the line. To determine chromosome congression, chromatids were counted as incorrectly aligned if they were outside of a rectangular area encompassing the central 30% of the spindle, a volume that generously encompasses wild-type metaphase plates. To determine this region accurately, we only considered cells aligned along the plane of the coverslips, where both spindle poles could be easily distinguished. Cells were considered misaligned when ≥1 kinetochore fell outside this central region, thick when kinetochores occupied >half of this volume and aligned when kinetochores occupied ≤half the volume. In Fig. 3c, we considered only misaligned (that is, falling outside the equatorial 30% volume) kinetochores and counted the number of these per condition.

For the statistical analysis for significance of FRAP data, the relationship between Bub1 WT, KD and T589A were compared among treatments by testing the equality of a set of parameters using F-tests, derived from the error sum of squares of the null model and the full model in SAS. All other statistical analysis was performed with Sigmaplot. Data are expressed as means±s.e.m. The data were analysed by ANOVA or *t*-tests (two-tailed) for determination of significance of the differences or as otherwise indicated. A *P*-value of <0.05 was considered to be statistically significant.

## Additional information

**How to cite this article:** Asghar, A. *et al.* Bub1 autophosphorylation feeds back to regulate kinetochore docking and promote localized substrate phosphorylation. *Nat. Commun.* 6:8364 doi: 10.1038/ncomms9364 (2015).

## Supplementary Material

Supplementary Figures Supplementary Figures 1-4

Supplementary Data 1 Bub1 phosphosites and autophosphorylation sites identified by SILAC and Mass spectrometry.

Supplementary Movie 1 HeLa stable cell lines expressing 3xMYC-GFP-tagged Bub1 WT were treated with Bub1 siRNA, transfected with an mRFP-H2B expressing plasmid, and synchronized in mitosis by release from a thymidine release 72 hours after transfection. The movie shows chromosome movement of a representative cell. The time stamp is in min:secs.

Supplementary Movie 2 HeLa stable cell lines expressing 3xMYC-GFP-tagged Bub1 KD were treated with Bub1 siRNA, transfected with an mRFP-H2B expressing plasmid, and synchronized in mitosis by release from a thymidine release 72 hours after transfection. The movie shows chromosome movement of a representative cell. The time stamp is in min:secs.

Supplementary Movie 3 HeLa stable cell lines expressing 3xMYC-GFP-tagged Bub1 T589A were treated with Bub1 siRNA, transfected with an mRFP-H2B expressing plasmid, and synchronized in mitosis by release from a thymidine release 72 hours after transfection. The movie shows chromosome movement of a representative cell. The time stamp is in min:secs.
