# Supplementary material for: Bub1 autophosphorylation feeds back to regulate kinetochore docking and promote localized substrate phosphorylation
Source: Nat Commun. 2015 Sep 24;6:8364. doi: 10.1038/ncomms9364 (PMC4598568; doi:10.1038/ncomms9364)
Supplement: Supplementary Figures — 1-4 [file ncomms9364-s1.pdf]

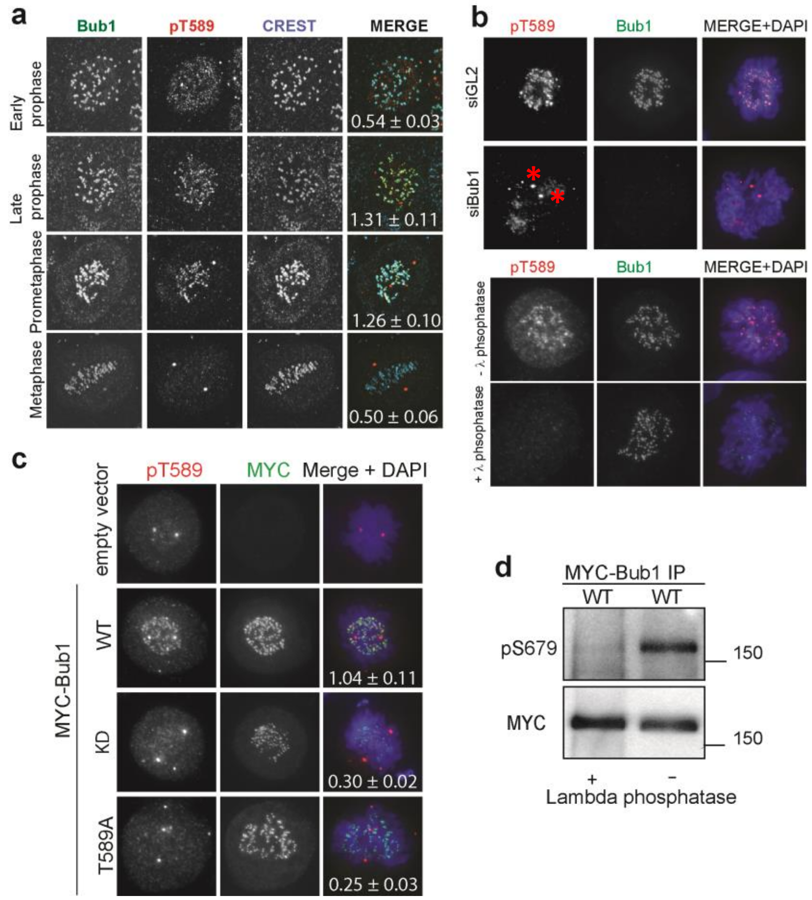

Supplementary Figure 1 Characterization of the pT589 and pS679 Bub1 antibodies. (**a**) Asynchronous HeLa S3 cells were fixed and stained with the Bub1 anti-pT589 antibody (red) as well as Bub1 (green) and CREST (blue). (**b**) Bub1-specificity (upper panels) and phosphospecificity (lower panels) of the Bub1 T589A antibody. Asterix indicates non-specific centrosomal staining that is not lost upon Bub1 depletion. (**c**) Cells depleted of endogenous Bub1 were rescued with either an empty vector plasmid, or plasmids carrying Bub1 WT, KD, T589A and synchronized in mitosis before fixation and immunofluorescence with anti-pT589 antibody (red) and MYC-Bub1 (green). DNA was counterstained with Hoechst (blue). (**d**) MYC-tagged Bub1-WT was expressed and immunoprecipitated from 293T cells, before the sample was equally divided and either treated with λ-phosphatase or buffer as a control. The immunoprecipitates were separated by SDS-PAGE and immunoblotted with the Bub1 anti-pS679 antibody (Upper panel) and reprobed with anti-MYC (bottom panel). For (**a**) and (**c**), the quantification in the MERGE panel represents the relative pT589 intensity normalized to CREST in arbitrary units. Scale bar= 5 μM.

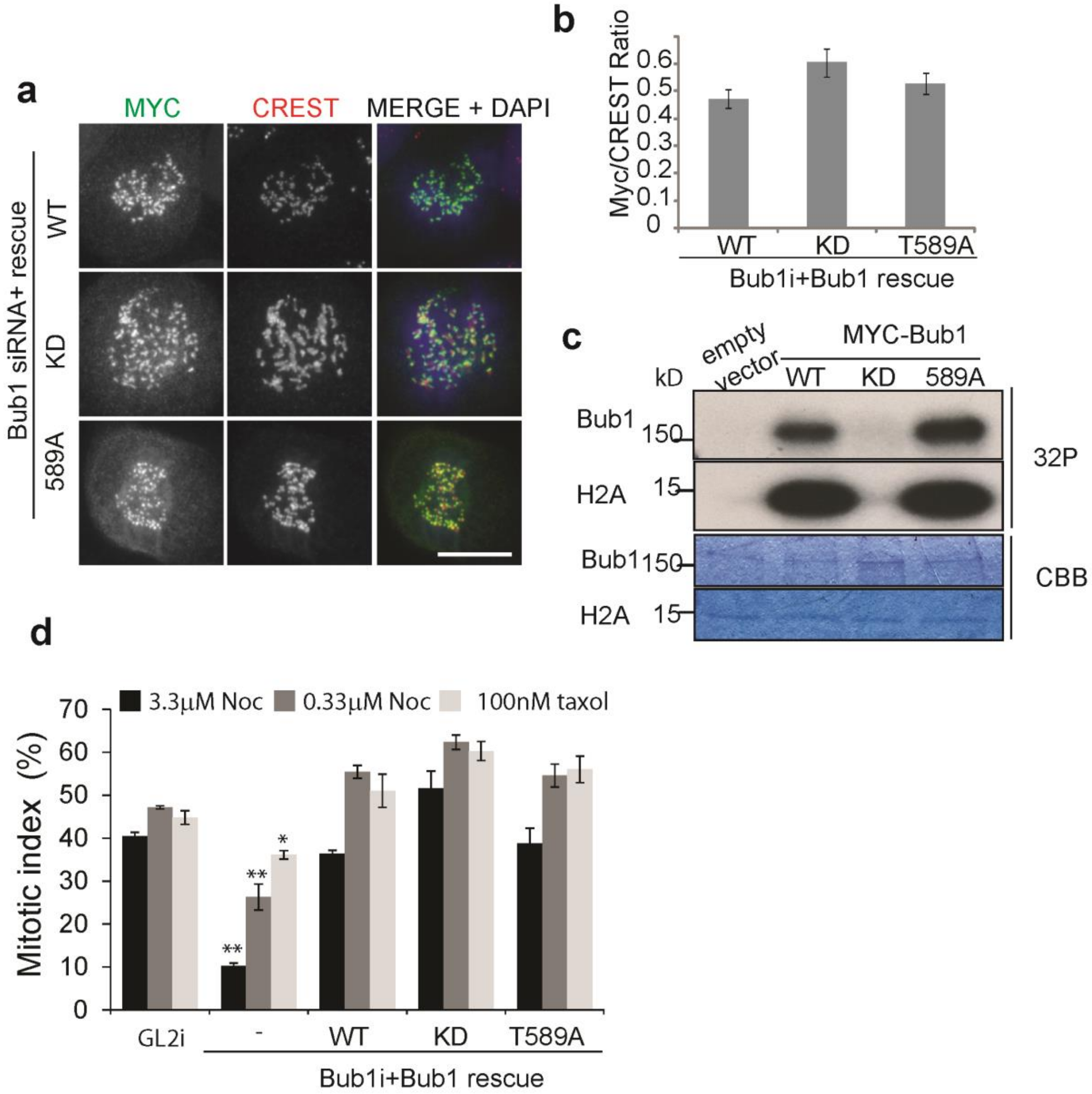

Supplementary Figure 2 Characterization of the isogenic MYC-GFP Bub1 WT, KD and T589A HeLa cell lines. (a) Immunofluorescenc of the 3XMYC-GFP Bub1-WT, KD, and T589A cell lines. Cells were stained with anti-MYC (green), anti-CREST (red) and Hoechst 33342 (blue) to mark the DNA. Scale bar= 10μM. (**b**) Quantitation of kinetochore fluorescence intensity of the MYC signal relative to CREST from (**a**), n=10 cells. Note the slightly higher kinetochore levels of Bub1-KD in the isogenic cell line which likely explains the slight increase in the mitotic index in (**d**) (**c**) *In vitro* kinase assay of Bub1-WT, KD, and T589A as well as an empty vector control. Cells were transfected with MYC-tagged Bub1-WT, KD, or T589A and the immunoprecipitated MYC-tagged Bub1 proteins were subjected to an *in vitro* kinase assay to monitor autophosphorylation of Bub1-WT, KD, and T89A (1st panel) as well their ability to phosphorylate histone H2A (2nd

Panel). Coommassie-stained gels to demonstrate protein loading are indicated below the autoradiograms (panels 3 and 4).(**d**) Bub1-WT, KD and T589A expressing cells or parental cells were depleted of endogenous Bub1 and treated overnight with nocodazole (Noc, 3.3 µM or 0.33µM) or 100nM taxol to arrest the cells. GL2 siRNA cells were used as controls. The mitotic index was counted in each condition. The data represent the mean ±SE of three independent experiments with 100-600 cells counted per condition. Significance was calculated by one-way ANOVA relative to the Bub1 siRNA condition. ** $p<0.001$, * $p<0.03$.

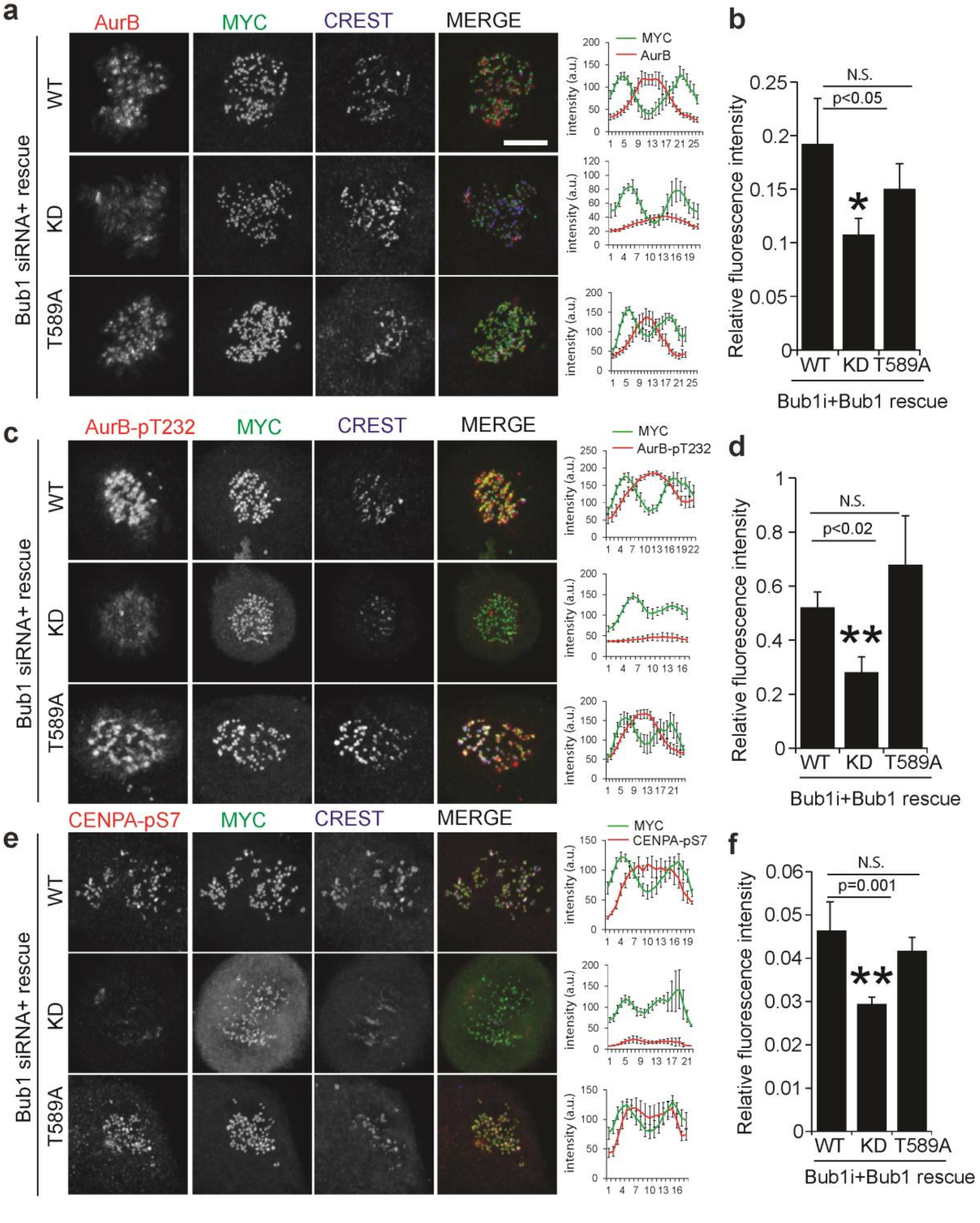

Supplementary Figure 3 Aurora recruitment and activation are normal in Bub1-T589A expressing cells. Bub1 WT, KD and T589A expressing cells were depleted of endogenous Bub1 and localization (**a,b**), phosphorylation of the activation loop T232 (**c,d**) and of the canonical substrate CENPA-S7 (**e,f**) were

verified by immunofluorescence. Signal profile across sister kinetochores showing signal presence and/or overlap is indicated to the right of each set of images and represent the mean profile ± SE of 7-15 kinetochore pairs. Quatitations (**b,d,f**) represent normalized kinetochore signals of 8-13 cells per condition. Error bars represent SE and significance was measures by the t-test. Scale bar = 5µM.

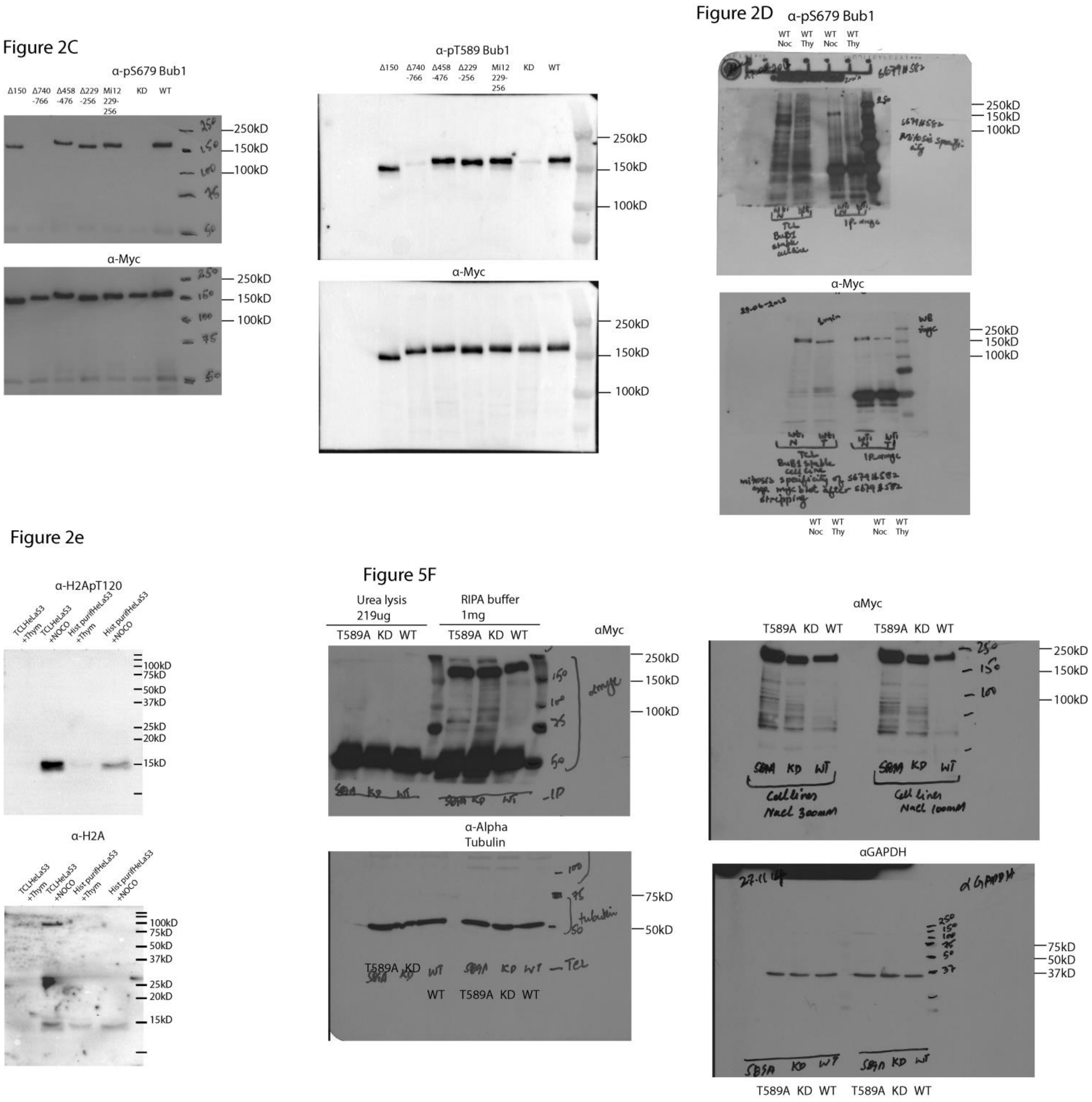

Supplementary Figure 4. Original non-cropped Western Blots presented in this manuscript. Figure labelling corresponds to the figure in the main manuscript.
